# Supplementary material for: Lysophosphatidic acid modulates CD8 T cell immunosurveillance and metabolism to impair anti-tumor immunity
Source: Nat Commun. 2023 Jun 3;14:3214. doi: 10.1038/s41467-023-38933-4 (PMC10239450; doi:10.1038/s41467-023-38933-4)
Supplement: Supplementary file 1 — Supplementary Information [file 41467_2023_38933_MOESM1_ESM.pdf]

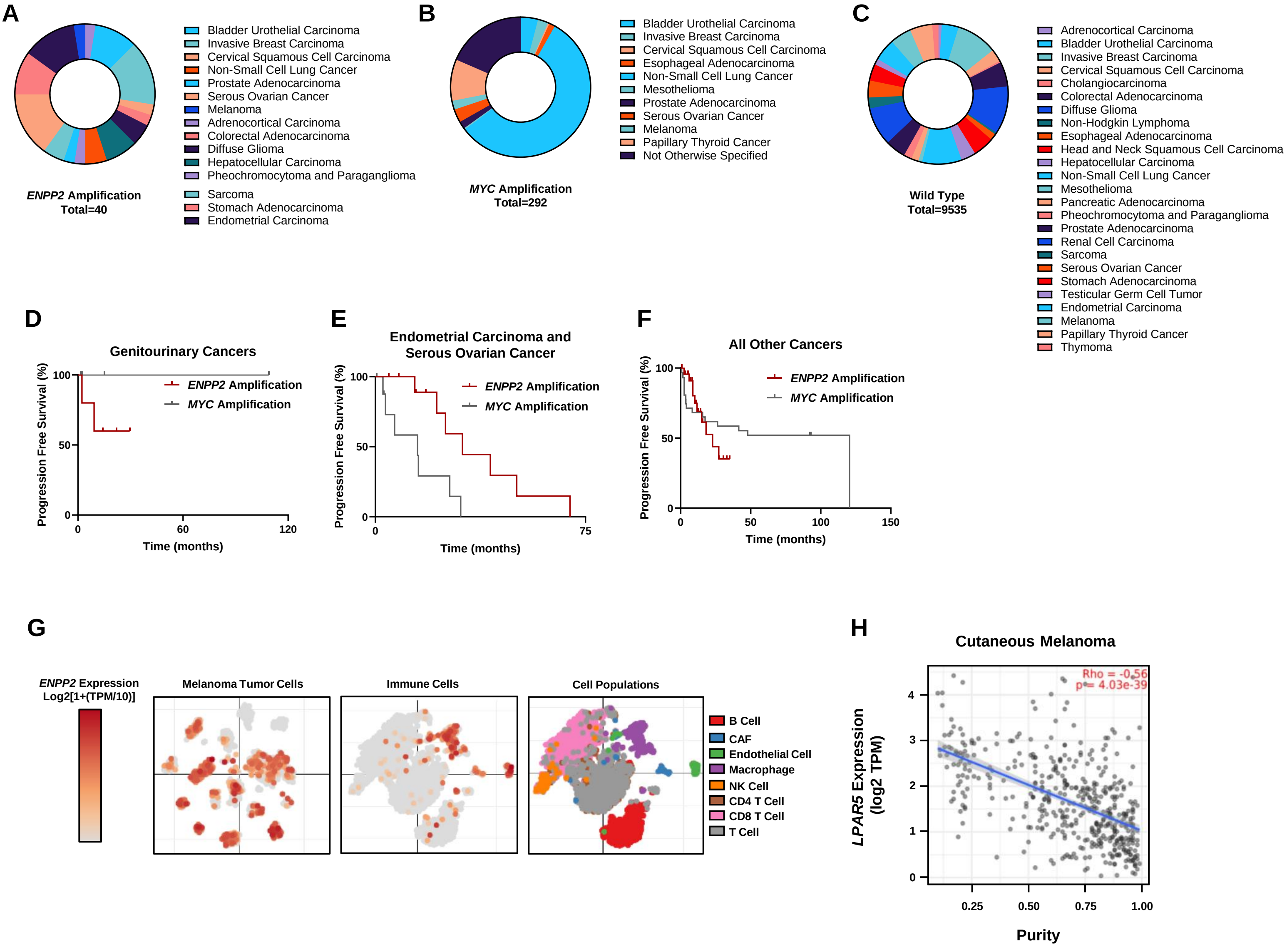

Supplementary Figure 1

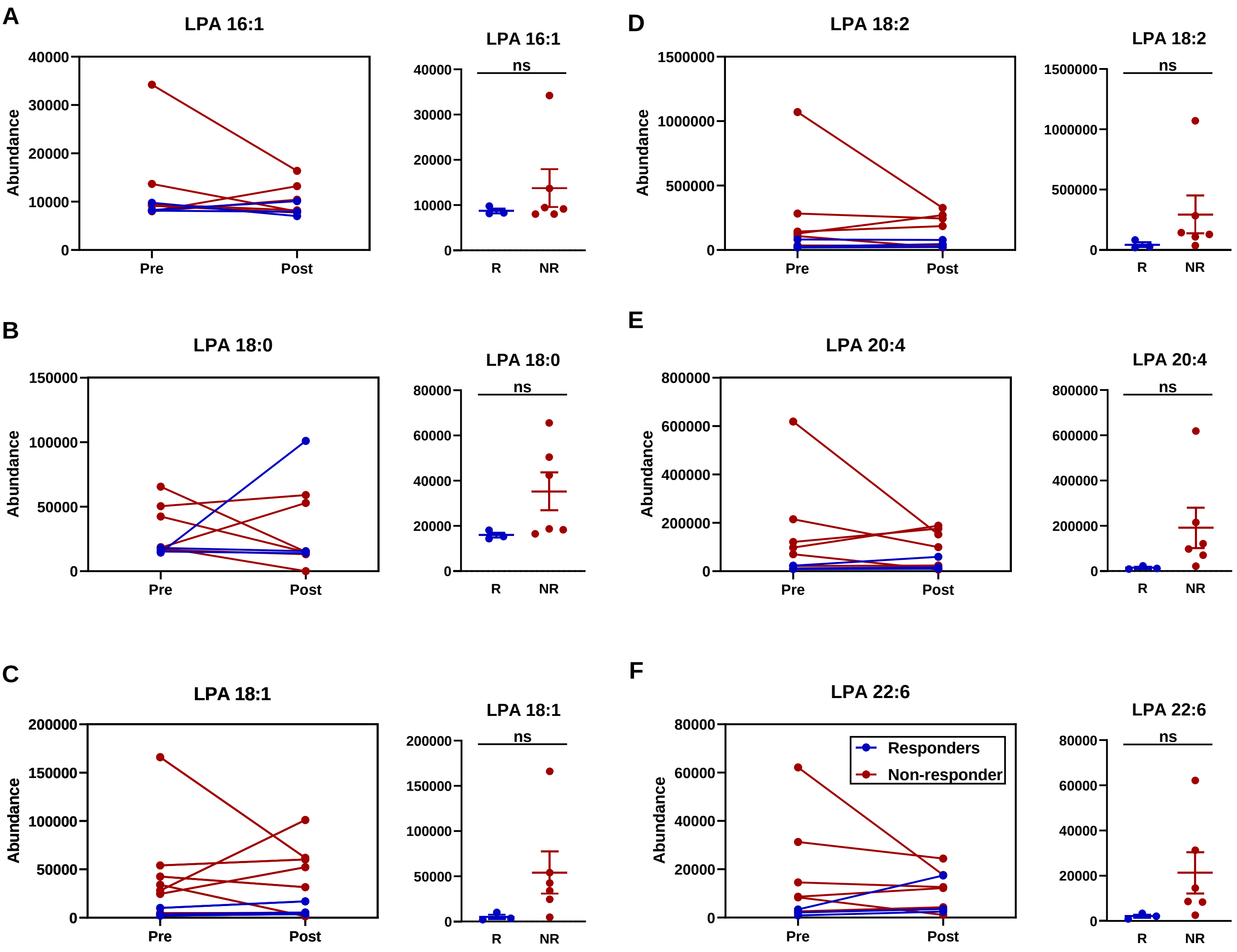

Supplementary Figure 2

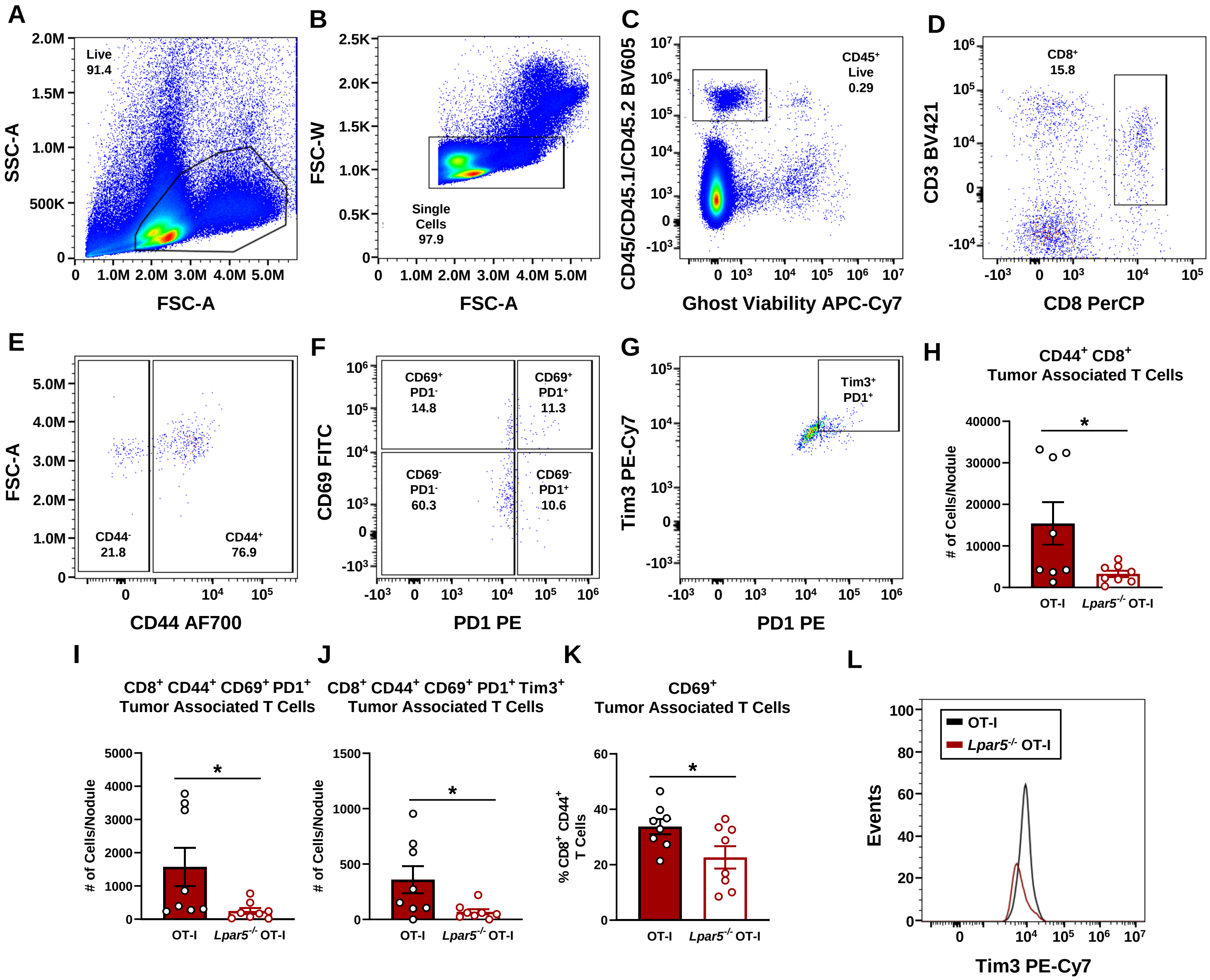

Supplementary Figure 3

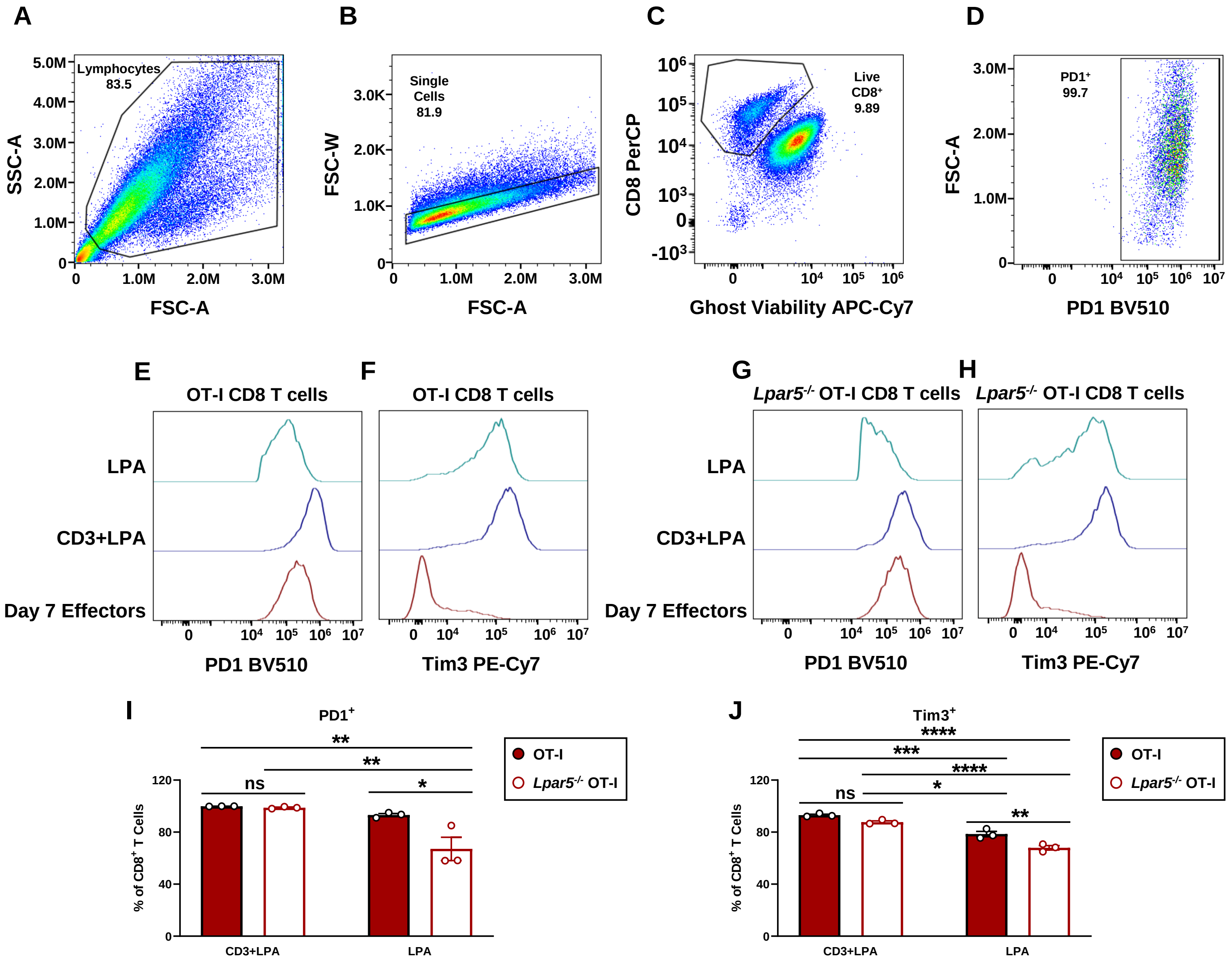

Supplementary Figure 4

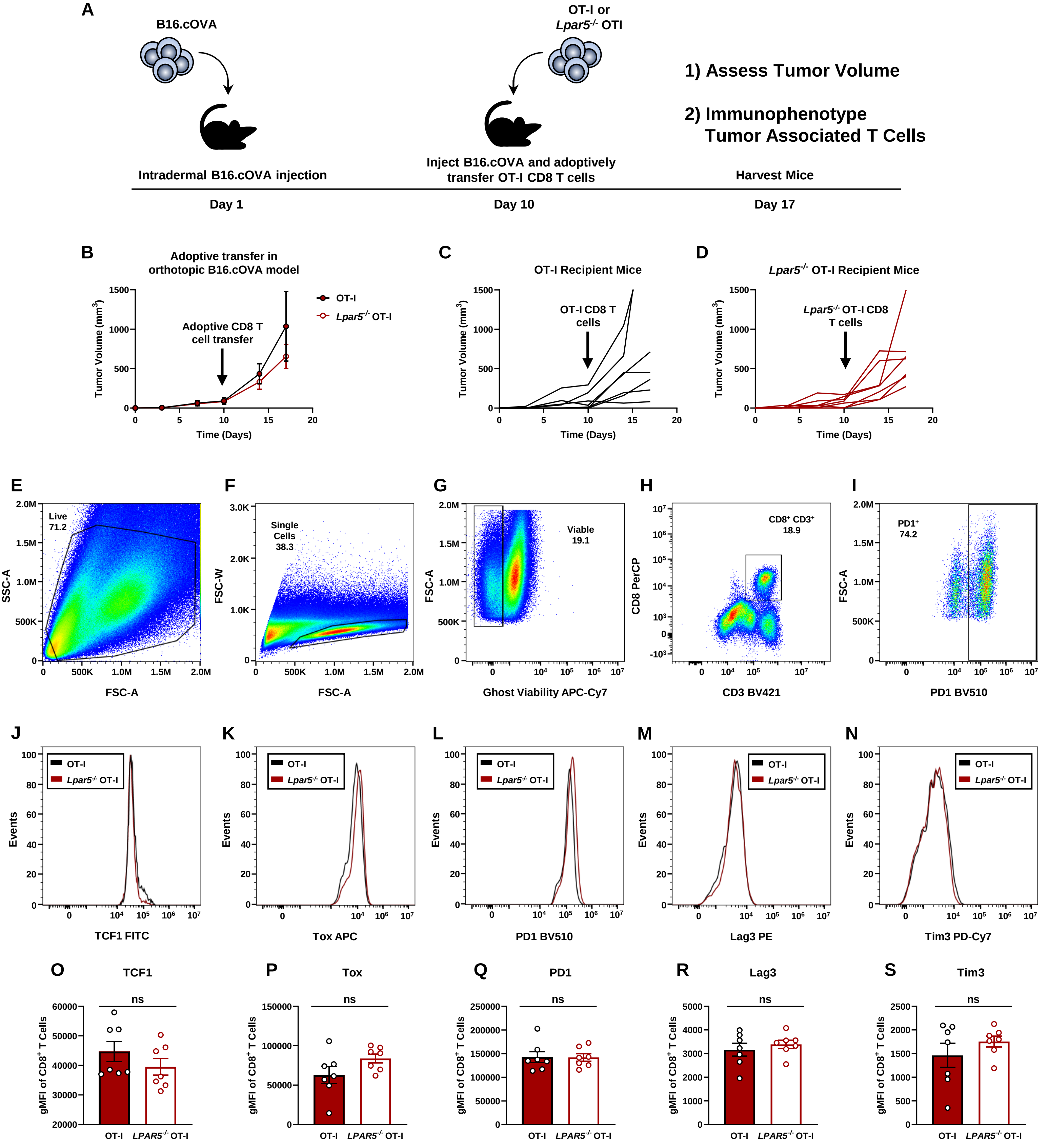

Supplementary Figure 5

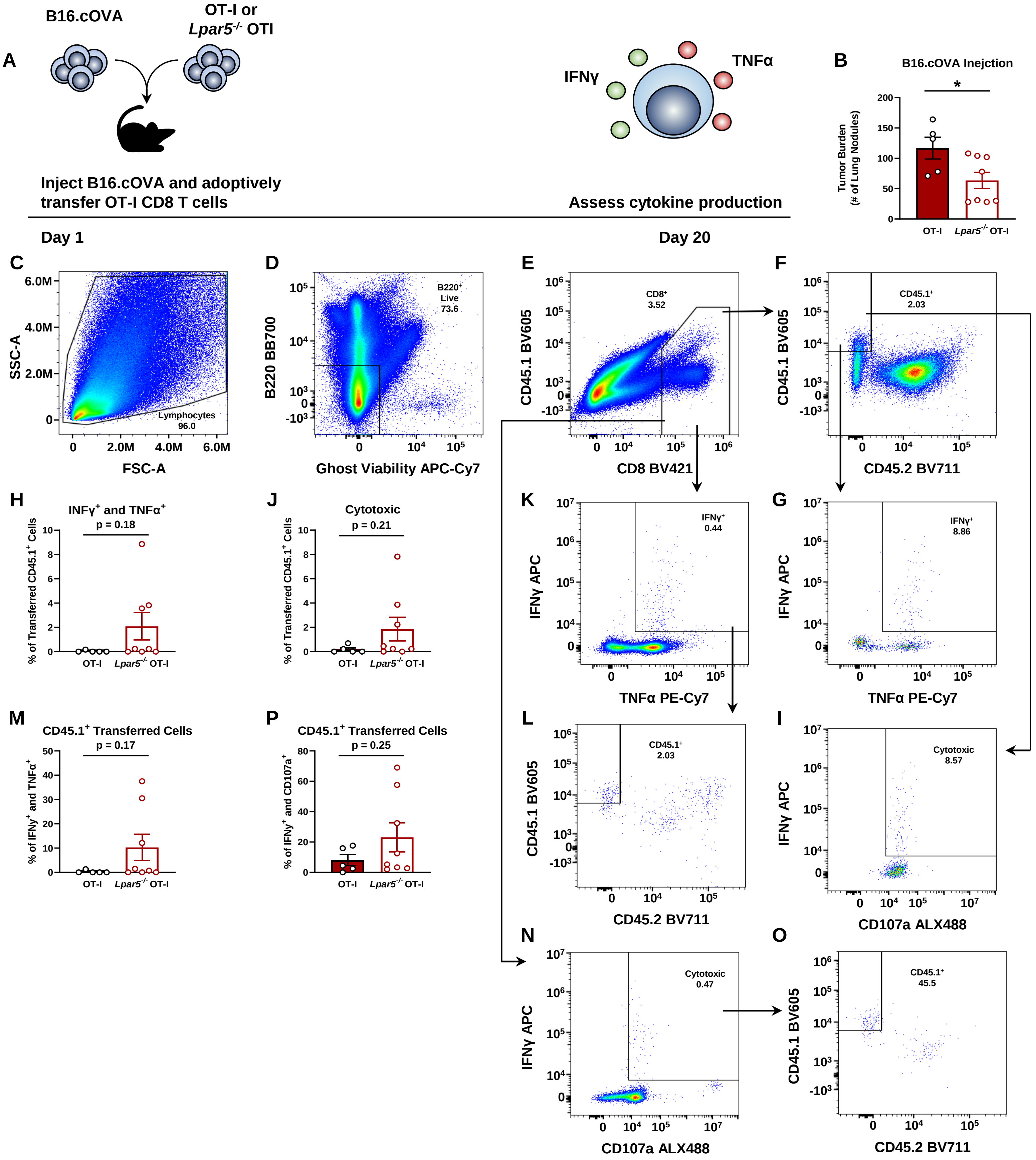

Supplementary Figure 6

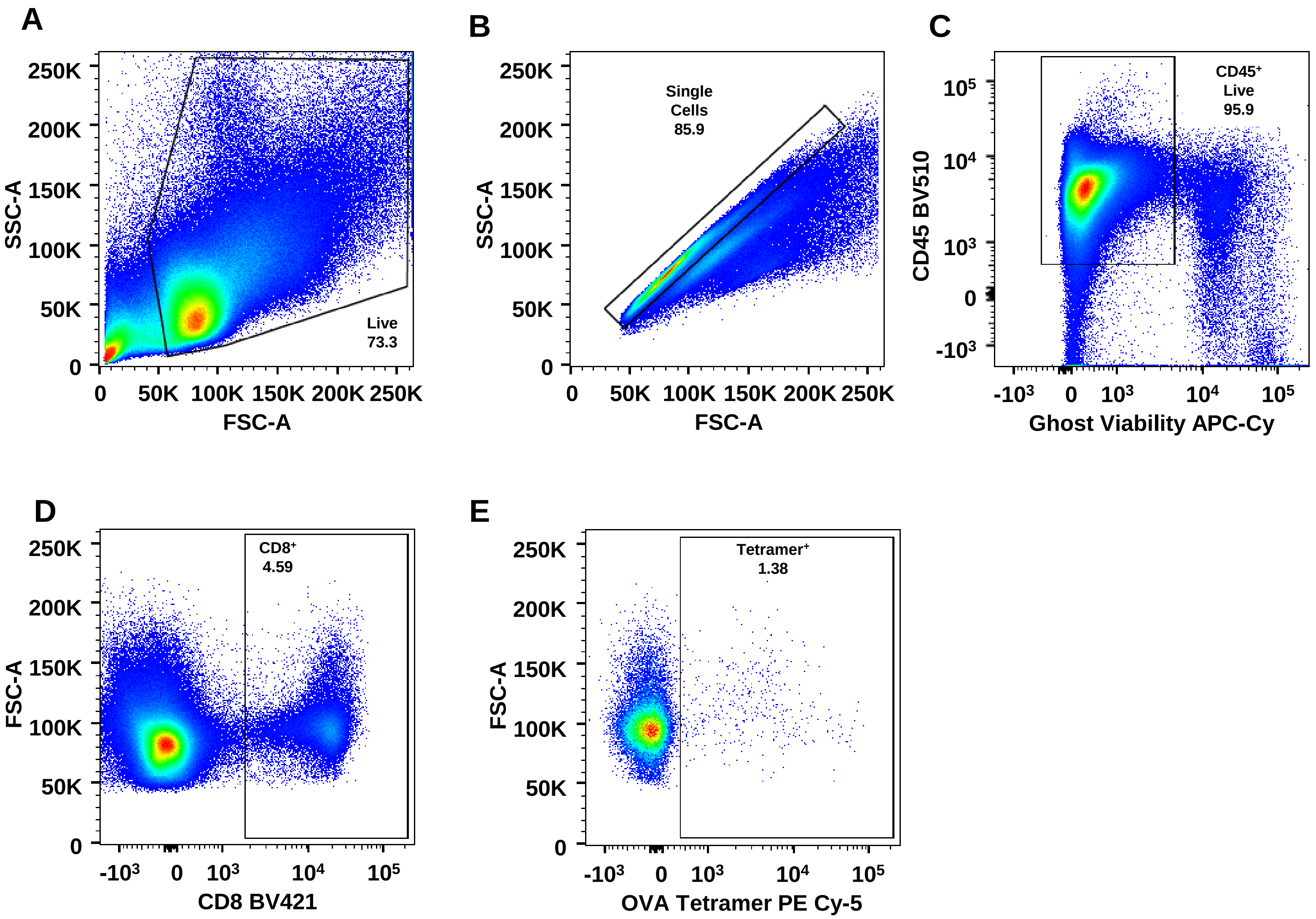

Supplementary Figure 7

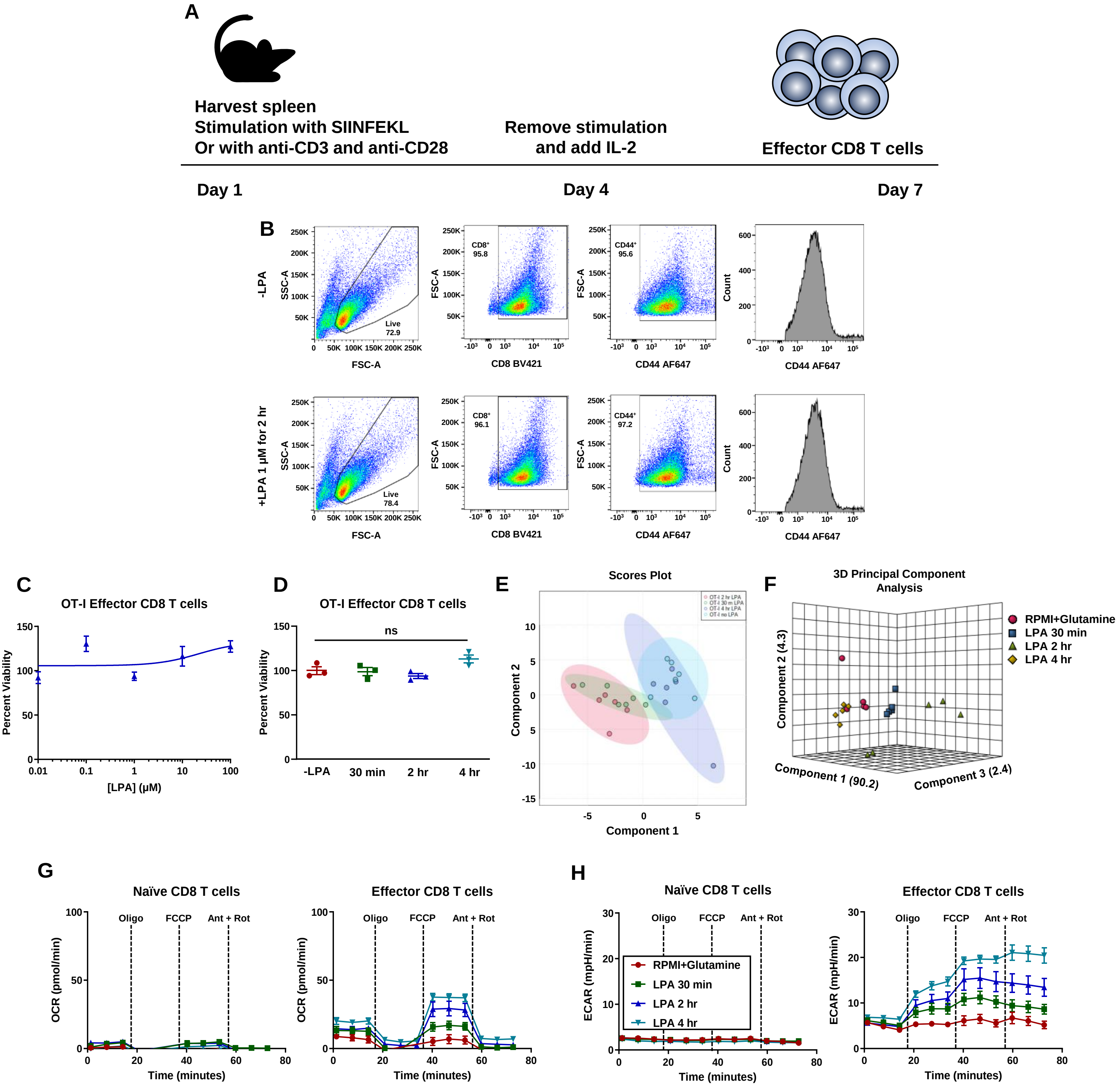

Supplementary Figure 8

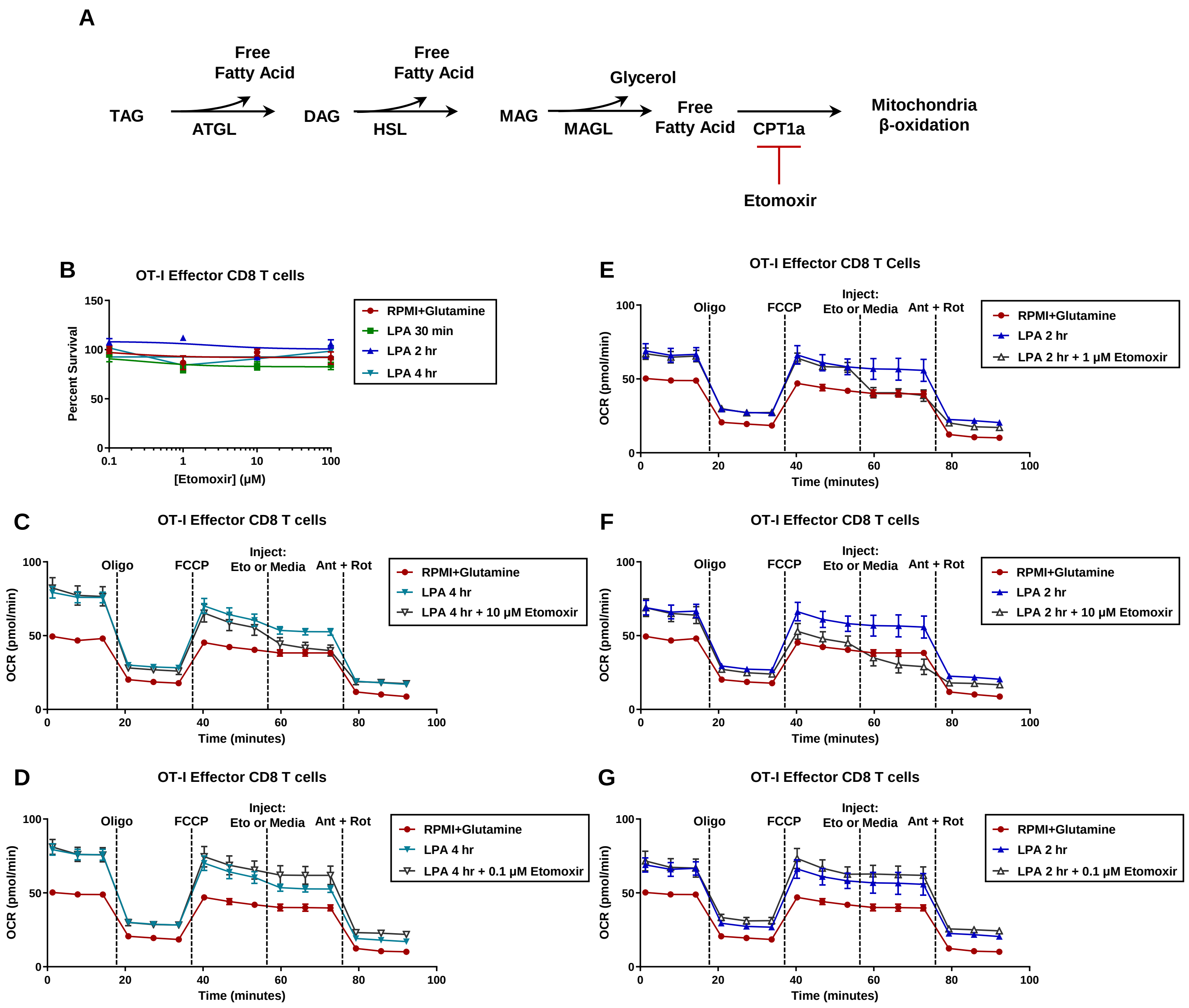

Supplementary Figure 9

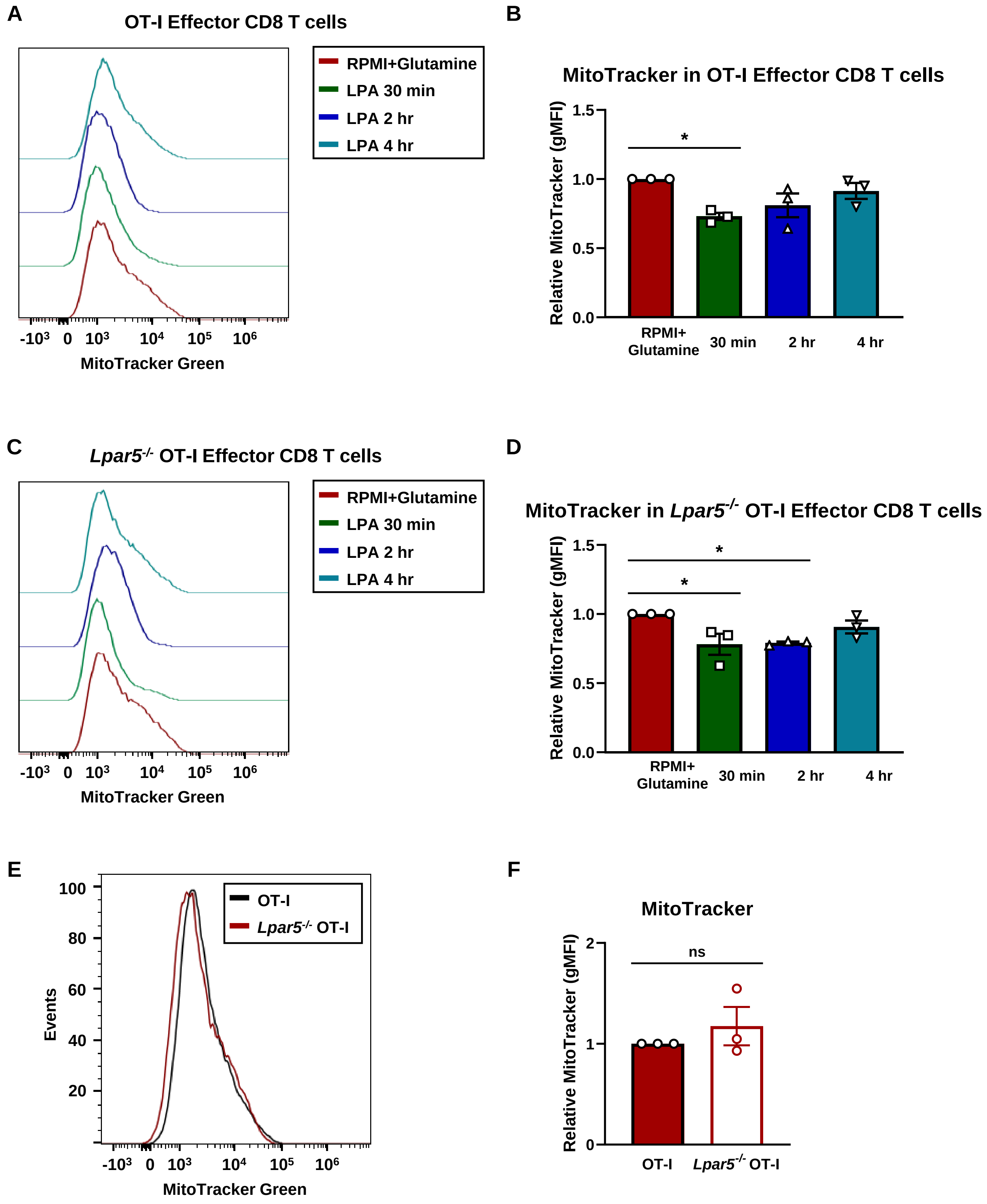

Supplementary Figure 10

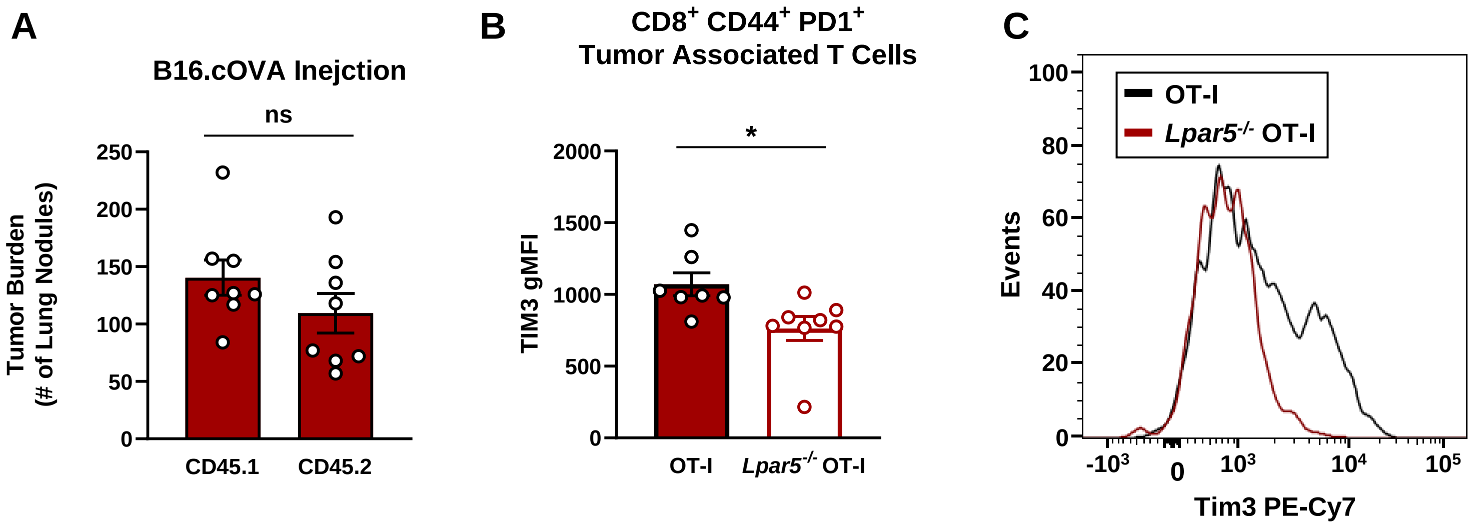

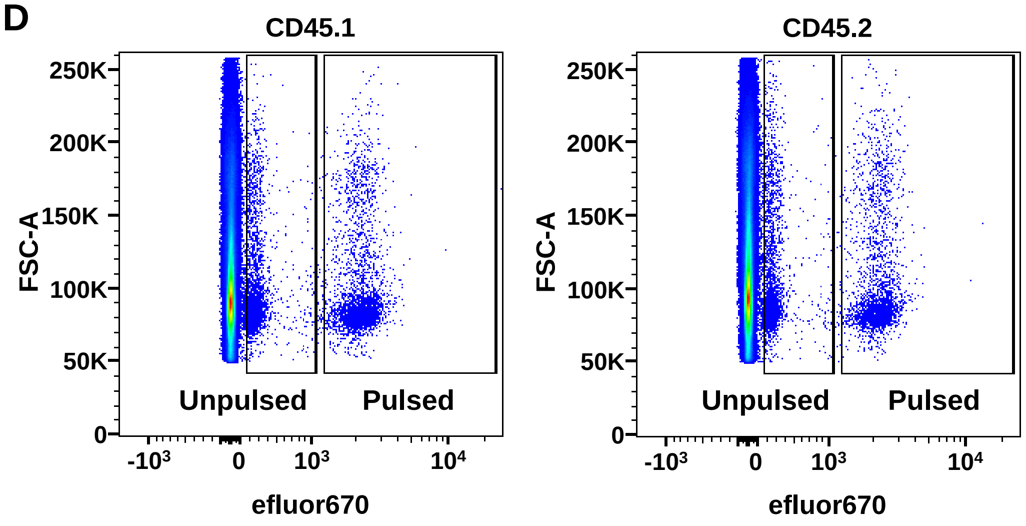

E

Antigen specific killing	
CD45.1	68.6%
CD45.2	70.7%

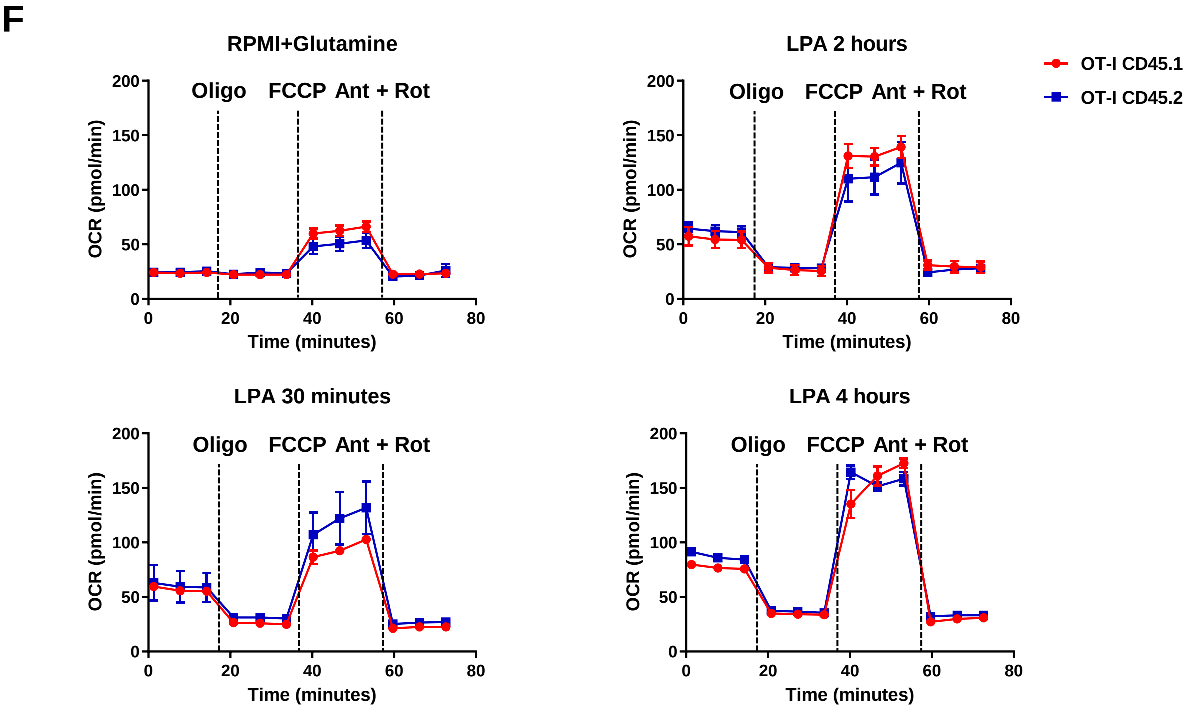

Supplementary Figure 11

Supplementary Methods

Melanoma Patient Samples

Melanoma Patient Samples

Blood samples from melanoma patients were collected from the University of Colorado Health Hospital and details of the collection are described in Supplemental Information. from 2008 to 2020. Plasma samples were collected in green-top heparin tubes with pre- and post-treatment samples collected from each patient. Samples were directly collected from the hospital, processed immediately, and stored at -80°C until analysis. Samples were collected under the Melanoma Biorepository at the University of Colorado Cancer Center. Patients were consented under approval from the Colorado Institutional Review Board (IRB# 05-0309). These patient studies were conducted in adherence according to the Declaration of Helsinki, Belmont Report, and U.S. Common Rule.

Flow Cytometry

Cells were stained in FACS/MACS buffer (PBS 2% FCS and sodium azide or EDTA). Fc receptor blocking was done with 2.4G2 (70-01610-M001, Invitrogen) on ice for 20 minutes. All cells were stained with live/dead staining with Ghost Red 780 (1:200 dilution factor, 13-0865-T100, Tonbo Biosciences). Cytometric analysis was conducted on either the LSRII flow cytometer (BD) or CytoFlex. Data were analyzed with FlowJo v8 (TreeStar).

In Vivo Tumor Models

For orthotopic tumor models, 2 million cells were intradermally injected onto the flanks of mice (n = 7 mice per group) on Day 1. Tumor volume and mouse weights were regularly measured throughout the course of the experiment. Mice were euthanized when tumors reached 1,500 mm^3. One million OT-I or *Lpar*$^{5-/-}$ OT-I CD8 T cells were adoptively transferred via intravenous injection on Day 10 and tumors were then harvested on Day 17. After mice were sacrificed, the flank tumors were dissected out, homogenized twice through cell strainers (100 µm, CELLTREAT Scientific Products), stained, and analyzed by flow cytometry. For the systemic tumor model in Figure 2 and Supplemental Figure 4, three to five tumors were dissected out, homogenized twice through cell strainers (100 µm, CELLTREAT Scientific Products), stained, and analyzed by flow cytometry. The remaining lung tissue was fixed using 10% formalin overnight then processed and embedded into formalin-fixed paraffin embedded (FFPE) blocks. For the systemic tumor model in Figure 3 and Supplemental Figure 7, lungs were harvested and tumors were quantified. Lungs were first finely chopped using scissors and then digested in a mix of 1 mL of collagenase/DNase at 37ºC for 30 minutes. During this incubation period, the lungs were shaken every 10 minutes. The digested mix was then pipetted and homogenized through cell strainers (100 µm, CELLTREAT Scientific Products). ACK lysis was performed in 1 mL of lysis buffer for 5 minutes at room temperature. Cells were either counted and stained for exhaustion markers or prepared for SIINFEKL stimulation to measure cytokines. Cells used for SIINFEKL stimulation ex vivo were run over a liquid percoll gradient (Cytiva). A 40/70 percoll gradient was prepared where 40% and 70% percoll were diluted from a 100% percoll into RPMI+FCS. The 100% percoll was prepared using a mix of stock percoll from Cytiva, 5x RPMI, and 7.5% sodium bicarbonate. The middle layer of the percoll represents the lymphocytes which was collected and counted and plated into a 96 well plate with 3 µg/mL of

Brefeldin A (Sigma-Aldrich) and CD107a (diluted at 1:00). Cells were plated triplicate to groups of: 1) unstimulated (media only), 2) SIINFEKL (stimulated with at 2 µg/ml), and 3) Phorbol 12-myristate 13-acetate (PMA stimulated at 50 ng/mL, Sigma-Aldrich, #P8139) and ionomycin (stimulated at 500 ng/mL, Sigma-Aldrich, #I9657). Cells were then incubated at 37ºC for 6 hours. After this incubation period, the cells were stained using the Ebioscience™ Foxp3 / Transcription Factor Staining Buffer Set (ThermoFisher Scientific) using MACS buffer (PBS with 0.5% BSA and 2mM EDTA).

Metabolic Flux Assay using the Seahorse MitoStress Test

The sensor cartridge was placed in a utility plate which was then loaded with 200 µL of XF Calibrant and hydrated in a non-CO_2 incubator overnight. Specifically, each well of the utility plate was filled with 200 µl of distilled water and the sensor cartridge was lowered onto the utility plate to ensure it was properly hydrated. The sensor was left in a non-CO_2 incubator at 37ºC overnight (minimum 10hrs). XF Calibrant was put in a 50 mL conical tube and also put in the non-CO_2 incubator with the sensor cartridge and utility plate overnight. Fresh Seahorse media was prepared the day before. Specifically, seahorse media was prepared with 10 mM glucose, 1 mM pyruvate, and 2 mM glutamine at 7.4 pH. On the day of, the distilled water was exchanged with XF calibrant. The distilled water was flicked out of the plate and 200 µl of calibrant was put in the utility plate with the sensor cartridge on top. The utility plate and sensor cartridge were then returned to the non-CO_2 incubator. CD8 T cells were prepared and plated at 200,000 cells per well in Seahorse media and cultured in a non-CO_2 incubator. In our assay set up, the CD8 T cells were plated and analyzed on the same day. Cocktails containing RPMI+Glutamine and LPA were prepared at x2 the concentration. The CD8 T cells were plated

in 90 µl of complete Seahorse media and then 90 µl of LPA-containing cocktails were added to the CD8 T cells and the LPA was diluted to 1 µM LPA on the microplate. Oligomycin (75351, Sigma-Aldrich, St. Louis, MO, USA), FCCP ((4-(trifluoromethoxy) phenyl) carbonohydrazonoyl dicyanide, C2920, Sigma), and antimycin A (A8674, Sigma-Aldrich) + rotenone (R8875, Sigma-Aldrich) were thawed on ice and prepared to be used at final concentrations of 2.5 µM, 2.0 µM, 0.5 µM, and 0.5 µM respectively (final concentrations of what is injected into the seahorse microplate at the time of the assay). Drugs were diluted into complete Seahorse media. Drugs were loaded into the sensor cartridge. Injection schemes are as follows: Port A) oligomycin, Port B) FCCP, and Port C) rotenone/antimycin or Port A) oligomycin, Port B) FCCP, Port C) etomoxir, and Port D) rotenone/antimycin. The CD8 T cells plated on the microplate were incubated in a non-CO_2 incubator 1 hour prior to loading the plate into the Seahorse instrument. The sensor cartridge was then loaded onto the Seahorse XFe96 Analyzer with 96-well plates (Agilent Technologies, Santa Clara, CA, USA) and pH and O_2 were assessed per well. Afterwards, the microplate containing CD8 T cells was then loaded and ran on the Seahorse XFe96 Analyzer. Etomoxir (E1905, Sigma-Aldrich) was titrated and acutely injected. All injected compounds were prepared in complete Seahorse media at a 7.4 pH and cells were analyzed for OCR and ECAR. Seahorse assay protocols were adjusted to perform acute injections of etomoxir (E1905 Sigma Aldrich). Etomoxir was loaded into the Seahorse port system and injected to a final concentration of 0.1, 1, or 10 µM. Treatment studies using the Lpar5 receptor antagonist, TC LPA5 4 (4708, Tocris), were performed as pre-treatment prior to running the Seahorse assay with 1 µM of TC LPA5 4. Analysis was performed using the Seahorse Wave software (Agilent Technologies, Santa Clara, CA, USA). Viability was assessed at the time of Seahorse using flow cytometric analysis.

Histology and Microscopy

FFPE blocks were cut into slides with 5 µm sections. FFPE tissue slides were deparaffinized and retrieved in preheated target retrieval solution, pH 9 (3-in-1, DAKO #K8004) at 96ºC for 20 minutes. Slides were removed after the PT link solution was cooled to 85ºC and transferred to room temperature diluted wash buffer for 5 minutes. Slides were stained on an automated Dako Link 48 Stainer, incubated for 10 minutes in dual endogenous enzyme block (Dako), and then incubated for 20 minutes in protein free blocking solution (Dako). Slides were then incubated in a dilution of 1:50 of CD8 (MS-457-S1, Thermo Fisher Scientific) primary antibody for 60 minutes at room temperature. Staining was developed with MACH2 Rabbit AP Polymer (BioCare Medical, Pacheco, CA, USA) for 30 minutes. Next, slides were incubated for 15 minutes in Vulcan Fast Red (BioCare Medical) and then washed with dH_2O. After each incubation step, the slides were washed using 1x wash buffer. Additionally, the slides were counterstained for 10 minutes with automated hematoxylin (Dako). Quantitative analyses were performed using ImageJ.

Viability Assays

Cell viability was assessed using the CellTiter Glo assay (Promega). Effector CD8 T cells were plated at 200,000 cells per well and given media without LPA (RPMI+Glutamine) or treated with LPA for 30 minutes, 2 hours, or 4 hours. Luminescence was read on the Synergy 2 plate reader (BioTek). Technical triplicates were analyzed for each condition and normalized to

no LPA treatment controls. Biological triplicates were performed, and technical error was propagated.

Statistical Analyses

Experiments were performed in biological replicates. Results will be expressed as mean ± standard error of the mean. Direct comparisons were made using non-parametric analyses, ANOVA, and Student’s t Test. Cohort sizes were determined based on statistical and power considerations.

Supplementary Figure Legends

Supplementary Figure 1. Assessing cancer types from TCGA harboring *ENPP2* amplification and tumor purity with *LPAR5* expression. (A,B,C) Graphic showing the distribution of cancer types from Figure 6 with *ENPP2* amplification where (A) represents tumors harboring an *ENPP2* amplification, (B) represents tumors harboring a *MYC* amplification, and (C) represents tumors that were wildtype for both *ENPP2* and *MY*C. Data demonstrate there are a spread of different cancer types with *ENPP2* amplification. (D,E,F) Progression free survival separated by cancer types (D) genitourinary, (E) endometrial and serous ovarian cancer, and (F) other cancers showing tumors that harbor either an *ENPP2* or *MYC* amplification. (G) *ENPP2* expression in melanoma and immune cells and corresponding immune cell populations. tSNE plots were generated using the Single Cell Portal (https://singlecell.broadinstitute.org/single_cell). (H) Correlation analysis of *LPAR5* expression and sample purity from cutaneous melanoma samples using TIMER2.0 (http://timer.cistrome.org/). Error bands for panel (H) represent the 95% confidence interval. The purity-adjusted spearman's rho = 0.56 and p = 4.03e-39.

Supplementary Figure 2. Paired and unpaired analysis of plasma lysophosphatidic acid from stage IV melanoma patients from Figure 6. (A,B,C,D,E,F) Lipid abundances were determined for LPA molecular species in patients pre- and post-therapy including (A) 16:1, (B) 18:0, (C) 18:1, (D) 18:2, (E) 20:4, and (F) 22:6. R = responder (n = 3 patients) and NR = non-responder (n = 6 patients). The two-sided unpaired and paired Student's t test analyses were performed where ns stands for not significant. Error bars for panels (A-F) represent standard error of the mean.

Supplementary Figure 3. Gating scheme and quantification of tumor associated T cells from Figure 2. (A,B,C,D,E) Tumor associated T cells were enumerated for after gating on (A) Live, (B) single cells, (C) $CD45^+$ Live (D) $CD3^+$ $CD8^+$ (E) $CD44^+$. (F,G) Cells were then further stratified based on exhaustion markers gating for either (F) $CD69^+$ $PD1^+$ or (G) $Tim3^+$ $PD1^+$. CD44, CD69, PD1, and Tim3 gates were determined using FMO gating. The panels presented represent a CD8 T cells from a mouse injected with *Lpar5*$^{-/-}$ OT-I CD8 T cells. (H,I,J) The number of tumor associated CD8 T cells per nodule was quantitated for (H) total $CD8^+$ $CD44^+$ where $p = 0.0337$, (I) $CD69^+$ $PD1^+$ where $p = 0.0382$, and (J) $Tim3^+$ $PD1^+$ where $p = 0.0353$ and $n = 8$ mice per group. (K) Percent of $CD69^+$ tumor associated T cells graphed from the percent of $CD45^+$ Live then $CD8^+$ $CD44^+$ where $n = 8$ mice per group and $p = 0.0391$. (L) Tim3 gMFI graphed from the percentage of cells from panel K. Statistics for this entire figure were performed using the unpaired two-sided Student's t test analysis was performed where * $p < 0.05$. Error bars for panels (H-K) represent standard error of the mean.

Supplementary Figure 4. Exhaustion markers on chronically stimulated cells. (A,B,C,D) Gating scheme for express more markers of exhaustion which show (A) lymphocytes, (B) single cells, (C) Live $CD8^+$ (D) $PD1^+$ cells. (E,F) Representative histograms of (E) PD1 and (F) Tim3 expression on unstimulated Day 7 effector OT-I CD8 T cells or chronically stimulated OT-I CD8 T cells with CD3, CD3+LPA, or LPA. (G,H) Representative histograms of (G) PD1 and (H) Tim3 expression on unstimulated Day 7 effector *Lpar5*$^{-/-}$ OT-I CD8 T cells or chronically stimulated *Lpar5*$^{-/-}$ OT-I CD8 T cells with anti-CD3+LPA or LPA. (I,J) Quantification of flow cytometric analysis of percent single positive (I) $PD1^+$ where exact p values are as follows, OT-I CD3+LPA vs *Lpar5*$^{-/-}$ OT-I LPA $p = 0.0039$; *Lpar5*$^{-/-}$ OT-I CD3+LPA vs *Lpar5*$^{-/-}$ OT-I LPA $p = 0.0048$; OT-I LPA vs *Lpar5*$^{-/-}$ OT-I LPA $p = 0.0149$, or (J) $Tim3^+$ where exact p values are as

follows OT-I CD3+LPA vs OT-I LPA p = 0.0004, OT-I CD3+LPA OT-I vs *Lpar5*$^{-/-}$ OT-I LPA p < 0.0001; *Lpar5*$^{-/-}$ OT-I CD3+LPA vs OT-I LPA p = 0.0100; *Lpar5*$^{-/-}$ OT-I CD3+LPA vs *Lpar5*$^{-/-}$ OT-I LPA p < 0.0001; OT-I LPA vs *Lpar5*$^{-/-}$ OT-I LPA p = 0.0043 from the CD8 T cell population where n = 3 mice per group. Statistics for this figure were performed using a Two-way ANOVA with Tukey's post-hoc analysis. Error bars for panels (I,J) represent standard error of the mean.

Supplementary Figure 5. (A) Experimental design schematic. (B,C,D) Adoptive CD8 T cell transfer in an orthotopic tumor model with (B) average tumor volume is shown or (C,D) individual tumors. Error bars represent standard error of the mean with n = 7 mice per group. (E,F,G,H,I) Gating scheme where tumors were harvested and gated on (A) Live, (B) Single Cells, (C) Viable, (D) $CD8^+CD3^+$, (E) $PD1^+$. (J,K,L,M,N) Representative histograms displaying (J) TCF1, (K) Tox, (L) PD1, (M) Lag3, (N) Tim3. (O,P,Q,R,S) Quantification of geometric mean fluorescence intensity (gMFI) of (O) TCF1, (P) Tox, (Q) PD1, (R) Lag3, (S) Tim3 where n = 7 mice per group. Statistics for this entire figure were performed using the unpaired two-sided Student's t test analysis was performed where * p < 0.05. Error bars for panels (O-S) represent standard error of the mean.

Supplementary Figure 6. Cytokine analysis on $CD45.1^+$ OT-I and *Lpar5*$^{-/-}$ OT-I CD8 T cells adoptively transferred into a systemic tumor model from Figure 3. (A) Experimental design schematic. (B) Quantified tumor burden in the lung after intravascular injection of B16.cOVA cells. Tumor burden is presented as the number of tumor nodules in the lung where n = 5 mice per OT-I group and n = 8 mice per *Lpar5*$^{-/-}$ OT-I group and p = 0.0347. (C,D,E,F,G) Gating scheme where cells were gated on (C) forward and side scatter (D) viable $B220^-$ cells (E) $CD8^+$ (F) $CD45.1^+$ and then (G) interferon γ^+ ($IFN\gamma^+$) and tumor necrosis factor α^+ ($TNF\alpha^+$) to identify

dual cytokine producing CD45.1$^+$ CD8 T cells. (H) Flow cytometric quantification for dual IFNγ$^+$ TNFα$^+$ as a percent of CD45.1$^+$ CD8 T cells where n = 5 mice per OT-I group and n = 8 mice per *Lpar5*$^{-/-}$ OT-I group. (I) Gating for cytotoxicity as measured by IFNγ$^+$ and CD107a$^+$ from the existing CD45.1$^+$ CD8 T cells. (J) Flow cytometric quantification for dual IFNγ$^+$ CD107a$^+$ as a percent of CD45.1$^+$ CD8 T cells n = 5 mice per OT-I group and n = 8 mice per *Lpar5*$^{-/-}$ OT-I group. (K,L) Gating scheme for (K) IFNγ$^+$ TNFα$^+$ off the CD8 T cell population then gated on (L) CD45.1$^+$ cells. (M) Flow cytometric quantification for CD45.1$^+$ CD8 T cells as a percent of IFNγ$^+$ TNFα$^+$ cells n = 5 mice per OT-I group and n = 8 mice per *Lpar5*$^{-/-}$ OT-I group. (N,O) Gating scheme for (N) cytotoxicity as measured by IFNγ$^+$ CD107a$^+$ off the CD8 T cell population then gated on (O) CD45.1$^+$ cells. (P) Flow cytometric quantification for CD45.1$^+$ CD8 T cells as a percent of IFNγ$^+$ CD107a$^+$ cells n = 5 mice per OT-I group and n = 8 mice per *Lpar5*$^{-/-}$ OT-I group. Statistics for this entire figure were performed using the unpaired two-sided Student's t test analysis was performed where * $p < 0.05$. Error bars for panels (B,H,J,M,P) represent standard error of the mean.

Supplementary Figure 7. Gating schematic for identifying tetramer$^+$ CD8 T cells after vaccination. Tetramer$^+$ CD8 T cells were enumerated in Figure 4 and first splenocytes were gated on (A) forward and side scatter (B) single cells (C) CD45$^+$ live (D) CD8$^+$ and then (E) Tetramer$^+$. Gating on Tetramer$^+$ CD8 T cells was determined based on fluorescence minus one (FMO) gating.

Supplementary Figure 8. Lysophosphatidic acid does not affect surface expression of CD8 and CD44, viability, or show obvious metabolic differences in CD8 T cells generated from anti-CD3 and anti-CD28 stimulation. (A) Generation of effector CD8 T cells ex vivo. Mice are harvested and CD8 T cells are activated with either SIINFEKL (N4) with other splenocytes serving as

antigen presenting cells or plate bound anti-CD3 and anti-CD28. The N4 or anti-CD3 and anti-CD28 are removed on Day 4 and replaced with IL-2. In the presence of IL-2, the CD8 T cells differentiate and expand into effectors which are used for flow cytometric analysis of day 7 effector CD8 T cells that are homogenous at this time after *in vitro* culture. (B) T cells are gated as lymphocytes, $CD8^+$, $CD44^+$. Expression of CD8 and CD44 is unchanged with LPA treatment with a single representative image shown. (C,D) Viability of day 7 effector CD8 T cells treated with (C) varying concentrations of LPA or (D) 1 µM LPA for 30 minutes, 2 hours, or 4 hours where n = 3 technical replicates per group. CD8 T cell viability is largely unchanged with the treatment of LPA. Statistical test using an ANOVA statistical test with post-hoc analysis was performed where * $p < 0.05$ and ns designates not statistically significant. (E,F) Dimension reduction analysis with (E) an unbiased scores plot and (F) 3D principal component analysis (PCA) on global metabolomics data where n = 6 mice per group. (G,H) Seahorse showing (G) oxygen consumption rate (OCR) and (H) extracellular acidification rate (ECAR) on C57BL/6 naïve CD8 T cells or day 7 effector CD8 T cells generated from anti-CD3 and anti-CD28 stimulation where n = 6 technical replicates per group. Similar to OT-I CD8 T cells, the naïve CD8 T cells are metabolically quiescent, and the effector CD8 T cells also display increased OCR and ECAR in response to LPA treatment. Error bars for panels (C,D,G,H) represent standard error of the mean.

Supplementary Figure 9. Carnitine palmitoyltransferase 1a inhibition abrogates LPA-induced metabolism. (A) Schematic of the lipolytic pathway showing the breakdown and oxidation of lipid droplets. Triacylglycerols (TAG) are found in lipid droplets and are cleaved by adipose triglyceride lipase (ATGL). The product, diacylglycerol (DAG) is further cleaved by hormone sensitive lipase (HSL) to generate monoacylglycerol (MAG). MAG is further cleaved by

monoacylglycerol lipase (MAGL) to remove the glycerol backbone. Free fatty acids are transported into the mitochondria for oxidation by carnitine palmitoyltransferase 1a (CPT1a). Translocation of free fatty acids into the mitochondria is the rate-determining step of β-oxidation. CPT1a is inhibited by etomoxir. (B) Day 7 Effector CD8 T cell viability in response to etomoxir where n = 3 technical replicates per group. (C,D,E,F,G) Oxygen consumption rate of day 7 OT-I Effector CD8 T cells treated with either RPMI+Glutmaine or LPA for (C,D) 4 hours or (E,F,G) 2 hours prior to starting the assay where n = 6 technical replicates. The assay was modified to inject either Seahorse media or etomoxir to a final concentration of 0.1, 1, or 10 µM. Error bars for panels (B-G) represent standard error of the mean.

Supplementary Figure 10. LPA induces a transient decrease in mitochondrial mass. (A,B) Flow cytometric analysis of LPA treatment on OT-I effector CD8 T cells with 1 µM LPA where (A) shows representative histograms and (B) is the quantitative analysis of n = 3 mice per group across three independent experiments where p = 0.0301. (C,D) Flow cytometric analysis of LPA treatment on *Lpar5*$^{-/-}$ OT-I effector CD8 T cells with 1 µM LPA where (C) shows representative histograms and (D) is the quantitative analysis across three independent experiments where RPMI+Glutamine vs 30 min LPA p = 0.0375 and RPMI+Glutamine vs 2 hr LPA p = 0.0469. (E,F) Flow cytometric analysis of LPA treatment on OT-I and *Lpar5*$^{-/-}$ OT-I effector CD8 T cells in the absence of LPA treatment where (E) shows a representative histogram and (F) is the quantitative analysis across three independent experiments. Statistics for this entire figure were performed using the unpaired two-sided Student’s t test analysis was performed where * $p < 0.05$. Error bars for panels (B,D,F) represent standard error of the mean.

Supplementary Figure 11. LPA phenotype persists in both CD45.1 and CD45.2 backgrounds. (A) Quantitated tumor burden (n = 8 mice per group) shows no statical difference between mice

that received CD45.1 versus CD45.2 OT-I CD8 T cells. (B,C) A reciprocal experiment of congenic markers in adoptive transfer shows that Tim3 expression remains statistically significant (n = 8 mice per group and panel C shows a representative histogram). Exact p value for panel (C) is as follows, $p = 0.0201$. (D,E) There is similar *in vivo* killing between CD45.1 and CD45.2 OT-I CD8 T cells. (D) shows representative dot plots and (E) shows percent specific killing from one mouse per each group. (F) CD45.1 and CD45.2 OT-I effector CD8 T cells respond similarly to LPA treatment where n = 6 technical replicates per group. Statistics for this entire figure were performed using the unpaired two-sided Student's t test analysis was performed where * $p < 0.05$. Error bars for panels (A,B,F) represent standard error of the mean.
